# Fast Algorithms for Structured Least Squares and Total Least Squares Problems

**DOI:** 10.6028/jres.111.010

**Published:** 2006-04-01

**Authors:** Anoop Kalsi, Dianne P. O’Leary

**Affiliations:** Applied Mathematics Program, University of Maryland, College Park, MD 20742; Dept. of Computer Science and Institute for Advanced Computer Studies, University of Maryland, College Park, MD 20742; National Institute of Standards and Technology, Gaithersburg, MD 20899-8910

**Keywords:** block Toeplitz matrix, displacement rank, errors in variables method, image deblurring, structured total least squares, Tikhonov regularization, total least squares

## Abstract

We consider the problem of solving least squares problems involving a matrix ***M*** of small displacement rank with respect to two matrices ***Z***_1_ and ***Z***_2_. We develop formulas for the generators of the matrix ***M*** *^H^****M*** in terms of the generators of ***M*** and show that the Cholesky factorization of the matrix ***M*** *^H^****M*** can be computed quickly if ***Z***_1_ is close to unitary and ***Z***_2_ is triangular and nilpotent. These conditions are satisfied for several classes of matrices, including Toeplitz, block Toeplitz, Hankel, and block Hankel, and for matrices whose blocks have such structure. Fast Cholesky factorization enables fast solution of least squares problems, total least squares problems, and regularized total least squares problems involving these classes of matrices.

## 1. Introduction

In [6], Mastronardi, Lemmerling, and Van Huffel present an algorithm for solving fast structured total least squares problems of the form

$$\underset{E,\beta ,x}{\min}{\left\|\left[\begin{array}{cc} E & \beta \end{array}\right]\right\|}_{F}^{2}$$ (1)

subject to the constraints

(A+E)x=y+β

with ***A*** ∈ *C^m^*^×^*^n^* a given Toeplitz matrix and ***y*** ∈ *C^m^*^×1^ a given vector. They include one additional constraint: ***E*** is a Toeplitz matrix. They produced a fast algorithm for solving this *structured total least squares problem* (STLS) and showed that the solution was a better estimator than the solution to the total least squares problem without the Toeplitz constraint.

In this paper, we work through the details of a small generalization of this algorithm. We consider the same problem (1), but under the constraint that ***A*** and ***E*** have small displacement rank relative to some matrices ***Z***_1_ and ***Z***_2_. Choosing these two matrices to be shift-down matrices and the rank to be two gives the Toeplitz constraint considered by [6], but we will be interested in other cases as well. See [3] for a discussion of displacement structure.

We also consider fast solution of the problem under the additional constraint that the norm of the solution vector ***x*** is specified, a problem posed in Pruessner and O’Leary [10]. This corresponds to a Tikhonov regularization of our structured total least squares problem and results in a fast solution algorithm for the problem considered in [8, 9, 10, 7].

The core of the algorithm in [6], based on a more general algorithm of [11], relies on two results: the representation of the generators for the matrix ***A****^H^****A*** that appears in the normal equations when ***A*** is Toeplitz, and then a fast factorization of a matrix derived from these generators. So we begin in Sec. 2 with a construction of the generators for ***M****^H^****M*** when ***M*** is any matrix of small displacement rank and ***Z***_1_ is close to unitary. In Sec. 3 we show that it is inexpensive to form a Cholesky factorization of ***M*** *^H^****M*** whenever ***Z***_2_ is triangular and nilpotent. Section 4 concerns the application of this algorithm, and we conclude in Sec. 5.

The results in this work are derived from [4, 5].

## 2. Generators of *M^H^M*

One way to solve a least squares problem

$$\underset{x}{\min}{\left\|\mathrm{Mx}-b\right\|}_{2}$$

involving a matrix ***M*** of full column rank is to use the normal equation formulation; we factor ***M****^H^****M*** = ***LL****^H^* where ***L*** is a square lower triangular matrix and then solve the linear system

$$LL^{H}x=M^{H}b.$$ (2)

Thus, we can solve this problem fast if we can compute a Cholesky factorization of ***M****^H^****M*** fast. To do this, we will first derive a generator for the matrix ***M****^H^****M*** when ***M*** ∈ *C^m^*^×^*^n^* (*m* ≥ *n*) has low displacement rank.

Suppose that ***M*** has low displacement rank relative to the matrices ***Z***_1_ ∈ *C^m^*^×^*^m^* and ***Z***_2_ ∈ *C^n^*^×^*^n^*, which means that if we define

$$N\equiv M-Z_{1}MZ_{2}^{H},$$

then rank (***N***), which we denote by *ρ*_1_, is small relative to *n*.

Suppose

$$\tilde{Z}=Z_{1}+W$$

is a unitary matrix $\left({\tilde{Z}}^{H}\tilde{Z}=I\right)$, where ***W*** has rank *ρ*_2_, also assumed to be small. For example, if ***E*** is Toeplitz, let ***Z***_1_ be the shift-down matrix with ones on its subdiagonal and zeros elsewhere, and then ***W*** is the matrix with a one in the last position of row 1.

Then ***M*** *^H^****M*** also has low displacement rank relative to ***Z***_2_, as we can see from the identity

$$\begin{array}{l} M^{H}M-Z_{2}M^{H}MZ_{2}^{H}=M^{H}M-Z_{2}M^{H}{\tilde{Z}}^{H}\tilde{Z}MZ_{2}^{H}\\ =M^{H}M-{\left(M-N+\mathrm{WM}Z_{2}^{H}\right)}^{H}\left(M-N+\mathrm{WM}Z_{2}^{H}\right)\\ ={\left(N-\mathrm{WM}Z_{2}^{H}\right)}^{H}\left(M-N+\mathrm{WM}Z_{2}^{H}\right)+M^{H}\left(N-\mathrm{WM}Z_{2}^{H}\right). \end{array}$$

**Theorem 1.** If the rank of $N\equiv M-Z_{1}MZ_{2}^{H}$ is *ρ*_1_ and if the unitary matrix $\tilde{Z}$ is equal to ***Z***_1_ + ***W*** where ***W*** has rank *ρ*_2_, then

$$\begin{array}{l} M^{H}M-Z_{2}M^{H}MZ_{2}^{H}=\\ -N^{H}N+N^{H}\left(M+\mathrm{WM}Z_{2}^{H}\right)+\left(M^{H}+{\left(\mathrm{WM}Z_{2}^{H}\right)}^{H}\right)N\\ -{\left(\mathrm{WM}Z_{2}^{H}\right)}^{H}\left(\mathrm{WM}Z_{2}^{H}\right)-M^{H}\left(\mathrm{WM}Z_{2}^{H}\right)-{\left(\mathrm{WM}Z_{2}^{H}\right)}^{H}M \end{array}$$

has rank at most 2(*ρ*_1_ + *ρ*_2_).

**Proof:** The equation in the statement of the theorem is a regrouping of the terms in the previous equation. The rank of $N-\mathrm{WM}Z_{2}^{H}$ is at most the rank of ***N*** plus the rank of ***W***, so the rank of the sum in that equation is at most 2(*ρ*_1_ + *ρ*_2_). []

An alternate proof of this theorem can be derived by noting that displacement rank is preserved by taking a Schur complement [3, Lemma 1.5.2], and ***M****^H^****M*** is the Schur complement of

$$\left[\begin{array}{cc} -I & M\\ M^{H} & 0 \end{array}\right]$$

which has displacement rank at most 2(*ρ*_1_ + *ρ*_2_) relative to the matrix

$$\left[\begin{array}{cc} Z_{1} & 0\\ 0 & Z_{2} \end{array}\right].$$

The bound 2(*ρ*_1_ + *ρ*_2_) can be an overestimate, since the ***N*** and ***W*** terms may interact. For example, for a Toeplitz matrix with rank computed relative to the shift-down matrices, the bound yields 6 while the actual rank is 4. If we use circular shifts, though, both the bound and the actual rank are 4.

Using the identity

$$ab^{H}+ba^{H}=\frac{1}{2}\left(a+b\right){\left(a+b\right)}^{H}-\frac{1}{2}\left(a-b\right){\left(a-b\right)}^{H},$$

## 3. Determining a Factorization of *M^H^M* From the Generators

Theorem 1 tells us how to determine *ρ* = 2 (*ρ*_1_ *+ ρ*_2_) vectors ***h****_i_* so that

$$M^{H}M-Z_{2}M^{H}MZ_{2}^{H}=\sum_{i=1}^{\rho }s_{i}h_{i}h_{i}^{H}$$ (3)

where *s_i_* equals plus or minus 1. When ***Z***_1_ and ***Z***_2_ are shift-down matrices, it has been shown [6, 1, 2] that this implies that

$$\begin{array}{l} M^{H}M=\sum_{i=1}^{\rho }s_{i}L\left(h_{i}\right)L{\left(h_{i}\right)}^{H}\\ \;=\left[L\left(h_{1}\right)\ldots L\left(h_{\rho }\right)\right]S\left[\begin{array}{c} L{\left(h_{1}\right)}^{H}\\ \vdots \\ L{\left(h_{\rho }\right)}^{H} \end{array}\right] \end{array}$$

where ***S*** = diag(***S****_i_*), ***S****_i_* = diag(*s_i_*) (*n × n*), and ***L***(***h****_i_*) is the lower triangular Toeplitz matrix with first column equal to ***h****_i_*. We now generalize this result somewhat.

### 3.1 Triangular Factors of Structured Matrices

**Theorem 2.**

If ***Z***_1_ is nilpotent, then

$$A-Z_{1}AZ_{2}^{H}=gh^{h}$$

and only if

$$A=L_{1}\left(g\right)L_{2}^{H}\left(h\right)$$

where

$$L_{i}\left(x\right)=\left[\begin{array}{cccc} x & Z_{i}x & \ldots & Z_{i}^{n-1}x \end{array}\right].$$

**Proof:** Suppose $A=L_{1}\left(g\right)L_{2}^{H}\left(h\right)$. Observe that

$$\begin{array}{l} L_{1}\left(g\right)L_{2}^{H}\left(h\right)=\left[\begin{array}{cccc} g & Z_{1}g & \ldots & Z_{1}^{n-1}g \end{array}\right]\left[\begin{array}{c} h^{H}\\ h^{H}\left(Z_{2}^{H}\right)\\ \vdots \\ h^{H}{\left(Z_{2}^{H}\right)}^{n-1} \end{array}\right]\\ \;=\sum_{j=0}^{n-1}Z_{1}^{j}gh^{H}Z_{2}^{j} \end{array}$$

and

$$Z_{1}L_{1}\left(g\right)L_{2}^{H}\left(h\right)Z_{2}^{H}=\sum_{j=0}^{n-1}Z_{2}^{j+1}gh^{H}Z_{2}^{j+1}$$

so, since $Z_{1}^{n}=0$, we conclude that

$$L_{1}\left(g\right)L_{2}^{H}\left(h\right)-Z_{1}L_{1}\left(g\right)L_{2}^{H}\left(h\right)Z_{2}^{H}=gh^{H}.$$

To prove the converse, suppose $A-Z_{1}AZ_{2}^{H}=gh^{H}$. Then, since

$$gh^{H}=L_{1}\left(g\right)L_{2}^{H}\left(h\right)-Z_{1}L_{1}\left(g\right)L_{2}^{H}\left(h\right)Z_{2}^{H},$$

we conclude that if $E=A-L_{1}\left(g\right)L_{2}^{H}\left(h\right)$, then

$$E=Z_{1}EZ_{2}^{H}.$$

Now since ***Z***_1_ is nilpotent, $Z_{1}^{\rho }=0$ for some *p* ≤ *n*. Therefore, $Z_{1}^{p-1}E=Z_{1}^{p}EZ_{2}^{H}=0$, and working backward in powers of ***Z***_1_, we see that $Z_{1}^{0}E=Z_{1}EZ_{2}^{H}=0$, so $A=L_{1}\left(g\right)L_{2}^{H}\left(h\right)$. []

The following corollary can be proved by finite induction.

**Corollary 1:** If ***Z***_1_ is nilpotent, then

$$A-Z_{1}AZ_{2}^{H}=\sum_{i=1}^{\rho }g_{i}h_{i}^{H}$$

if and only if

$$A=\sum_{i=1}^{\rho }L_{1}\left(g_{i}\right)L_{2}^{H}\left(h_{i}\right).$$

### 3.2 Triangular Factors of *M^H^M*

In order to solve our least squares problem, we wish to determine a Cholesky factorization

$$M^{H}M=LL^{H},$$

so we need to reduce the matrix

$$\left[\begin{array}{c} L_{2}{\left(h_{1}\right)}^{H}\\ \vdots \\ L_{2}{\left(h_{\rho }\right)}^{H} \end{array}\right]$$

to upper triangular form, where the vectors ***h****_i_* are defined in (3).

If ***Z***_1_ and ***Z***_2_ are shift-down matrices, then [6] shows how to do this reduction fast. Using Corollary 1, we will see that this can be done fast whenever ***Z***_2_ is triangular and nilpotent. We present the algorithm for the case ***Z***_2_ lower triangular; the upper triangular case is analogous but works with the columns in reverse order.

The algorithm proceeds by columns, putting zeros below the main diagonal. Note that

$$\hat{L}\equiv \left[\begin{array}{c} L_{2}{\left(h_{1}\right)}^{H}\\ \vdots \\ L_{2}{\left(h_{\rho }\right)}^{H} \end{array}\right]=\left[\begin{array}{l} h_{1}^{H}\\ h_{1}^{H}Z_{2}^{H}\\ \ldots \\ h_{1}^{H}{\left(Z_{2}^{H}\right)}^{n}\\ \vdots \\ h_{\rho }^{H}\\ h_{\rho }^{H}Z_{2}^{H}\\ \begin{array}{l} \ldots \\ h_{\rho }^{H}{\left(Z_{2}^{H}\right)}^{n} \end{array} \end{array}\right].$$

Suppose we determine a rotation between the first row $h_{1}^{H}$ and row *n* + 1, which contains $h_{2}^{H}$, to zero the first element of $h_{2}^{H}$. The same rotation between $h_{1}^{H}{\left(Z_{2}^{H}\right)}^{j}$ and $h_{2}^{H}{\left(Z_{2}^{H}\right)}^{j}\left(j=1,\ldots ,n-1\right)$ also zeroes the first element of $h_{2}^{H}{\left(Z_{2}^{H}\right)}^{j}$ since $Z_{2}^{H}$ is upper triangular. Therefore, by introducing one zero into our matrix, we have implicitly introduced *n* − 1 more, so we can put zeroes below the main diagonal in column 1 by using only *ρ* − 1 rotations, independent of the size of *n*.

We then use the resulting second row, equal to the first row postmultiplied by $Z_{2}^{H}$, to zero the second element of row *n* + 1. Again this implicitly introduces additional zeros, *n* − 2 of them, and we complete the operations on column 2 by using *ρ* − 1 rotations.

If we repeat this for each column, we accomplish our reduction. Let ***H*** be the *ρ* × *n* matrix whose rows are $h_{i}^{H}$. We can thus reduce $\hat{L}$ to upper triangular form just by operating on the matrix ***H***.

We design our algorithm to use Givens rotations as often as possible, minimizing the number of hyperbolic rotations in order to preserve stability. A Givens rotation can be used between row *i* and row *j* whenever *s_i_* and *s_j_* (see equation (3)) have the same sign; if they have different signs, then we must use a hyperbolic rotation. We’ll assume that we have ordered the generators so that the first $\hat{\rho }$ rows of ***H*** have *s_i_* = 1 and the remaining ones have *s_i_* = − 1.

**Table:** **Algorithm Reduce** (***H***)^3^

For *j* = 1, …, *n*,
For *i* = 2, …, $\hat{\rho }$,
If *h_ij_* is nonzero, then
zero it by a Givens rotation between row 1 and row *i*;
end for
For $i=\hat{\rho }+2,\ldots ,\rho$,
If *h_ij_* is nonzero, then
zero it by a Givens rotation between row $\hat{\rho }+1$ and row *i*;
end for
If $h_{\hat{\rho }+1,j}$ is nonzero, then
zero it by a stabilized hyperbolic rotation between row 1 and row $\hat{\rho }+1$;
Then the *j*th row of ***L****^H^* is $h_{1}^{H}$, the first row of the current ***H*** matrix.
Replace the first row of ***H*** by $h_{1}^{H}\;Z_{2}^{H}$ to form the pivot row for the next value of *j*.
end for

The cost of this reduction is at most *O*(*ρn*^2^), ignoring sparsity, plus the cost of the multiplications by ***Z***_2_. Sometimes sparsity can reduce this cost significantly [7]. Without exploiting the structure of $\hat{L}$ the cost would be *O*(*ρn*^3^). Once the factors ***LL****^H^* are computed, they can then be used to solve (2).

## 4. Some Applications

Our fast algorithm enables us to solve least squares, total least squares, and regularized total least squares problems involving matrices for which ***Z***_1_ is close to unitary and ***Z***_2_ is triangular and nilpotent. This includes several important classes of structured matrices.

### 4.1 Solving Least Squares Problems

Consider first the least squares problem

$$\underset{x}{\min}{\left\|\mathrm{Mx}-b\right\|}_{2}$$

where ***M*** ∈ *C ^m^*^×^*^n^* has full column rank. We can use our algorithm if

- ***M*** is Toeplitz. Then ***Z***_1_ and ***Z***_2_ are the shift-down matrices of appropriate size and the displacement rank is 2.
- ***M*** is block Toeplitz:
  $$M=\left[\begin{array}{llll} M_{\delta } & M_{\delta -1} & \ldots & M_{1}\\ M_{\delta +1} & M_{\delta } & \ldots & M_{2}\\ \ldots & \ldots & \ldots & \ldots \\ M_{\delta +\gamma -1} & M_{\delta +\gamma -2} & \ldots & M_{\delta } \end{array}\right],$$
  where ***M****_δ_* has dimension *m* / *γ* × *n* / *δ*. Then choosing ***Z***_1_ ∈ *C^m^*^×^*^m^* as
  $$Z_{1}=\left[\begin{array}{lllll} 0 & 0 & \ldots & 0 & 0\\ I & 0 & \ldots & 0 & 0\\ \ldots & \ldots & \ldots & \ldots & \ldots \\ 0 & 0 & \ldots & I & 0 \end{array}\right],$$
  where each block has dimension *m/γ*×*m/γ*, and choosing ***Z***_2_∈*Cm*×*n* similarly but with blocks of dimension *n* / *δ* × *n* / *δ* gives a displacement rank of *m* / *γ* + *n* / *δ*.
- ***M*** has Toeplitz blocks:
  $$M=\left[\begin{array}{llll} M_{11} & M_{12} & \ldots & M_{1,\delta }\\ M_{21} & M_{22} & \ldots & M_{2,\delta }\\ \ldots & \ldots & \ldots & \ldots \\ M_{\gamma ,1} & M_{\gamma ,2} & \ldots & M_{\gamma ,\delta } \end{array}\right],$$
  where each block ***M****_ij_* is Toeplitz. Then choosing ***Z***_1_ ∈ *C^m^*^×^*^m^* as a block-diagonal matrix consisting of shift-down matrices of dimensions matching the column dimension of the diagonal blocks of ***M*** and choosing ***Z***_2_ ∈ *C^n^*^×^*^n^* similarly but with blocks matching the row dimension gives a displacement rank of 2(*γ* + *δ*).
- ***M*** Hankel, block Hankel, or having Hankel blocks. These cases are analogous to those considered above.

### 4.2 Solving Structured Total Least Squares Problems

In some cases the matrix of the problem can be estimated only with error, and we need to determine not just the parameters ***x*** of the least squares fit but also the corrections to the operator. This problem can be formulated as

$$\underset{E,\beta ,x}{\min}{\left\|\left[\begin{array}{cc} E & \beta \end{array}\right]\right\|}_{F}^{2}$$ (4)

subject to the constraints

(A+E)x=y+β

with ***A*** and ***E*** having the same structure.

Suppose that the matrix ***E*** can be specified by *p* parameters *α*_1_,…, *α_p_*. For example, if *E* is a Toeplitz matrix, then

$$E=\left[\begin{array}{llll} \alpha_{n} & \alpha_{n-1} & \ldots & \alpha_{1}\\ \alpha_{n+1} & \alpha_{n} & \ldots & \alpha_{2}\\ \ldots & \ldots & \ldots & \ldots \\ \alpha_{m+n-1} & \alpha_{m+n-2} & \ldots & \alpha_{m} \end{array}\right],$$

and *p* = *m* + *n* − 1. We rewrite our problem as

$$\underset{\alpha ,\beta ,x}{\min}{\left\|\left[\begin{array}{c} \beta \\ \alpha \end{array}\right]\right\|}_{2}^{2}$$ (5)

where

β=(A+E)x−y.

Following [6], we have replaced the term ${\left\|E\right\|}_{F}^{2}$ by ***α****^H^****α***, equivalent except for scaling of the entries $\alpha_{\iota }^{2}$.

We define the matrix ***X*** ∈ *C^m^*^×^*^p^* by the equation

Xa=Ex.

For example, if ***E*** is Toeplitz, then *p* = *m* + *n* − 1 and

$$X=\left[\begin{array}{llllllll} x_{n} & x_{n-1} & \ldots & x_{1} & 0 & \ldots & \ldots & 0\\ 0 & x_{n} & x_{n-1} & \ldots & x_{1} & 0 & \ldots & 0\\ \ldots & \ldots & \ldots & \ldots & \ldots & \ldots & \ldots & \ldots \\ 0 & \ldots & 0 & x_{n} & x_{n-1} & \ldots & \ldots & x_{1} \end{array}\right].$$

Following [11], we form a quadratic approximation to (5) by using linear approximations ***α*** + ***∆α*** and ***x*** + ***∆x***, resulting in

$$\begin{array}{l} \beta \approx (A+(E+\Delta E))(x+\Delta x)-y\\ \;\approx (A+E)x+X\Delta \alpha +(A+E)\Delta x-y \end{array}$$

so that

$${\left\|\left[\begin{array}{c} \beta \\ \alpha \end{array}\right]\right\|}_{2}^{2}={\left\|\left[{}_{I}^{X}{\;}_{0}^{A+E}\right]\left[\begin{array}{c} \Delta \alpha \\ \Delta x \end{array}\right]+\left[{}_{\alpha }^{\left(A+E\right)x-y}\right]\right\|}_{2}^{2}.$$

If we minimize this with respect to ***∆α*** and ***∆x***, then we can form a new approximation

$$\begin{array}{l} \alpha =\alpha +\Delta \alpha \\ x=x+\Delta x \end{array}$$

to the solution of (5) and then repeat the procedure until convergence. As noted by [11], this is a Gauss-Newton algorithm applied to (5). Therefore, the main computational task is to solve linear least squares problems of the form

$$\underset{\Delta \alpha ,\Delta x}{\min}{\left\|M\left[\begin{array}{c} \Delta \alpha \\ \Delta x \end{array}\right]+\left[\begin{array}{c} (A+E)x-y\\ \alpha \end{array}\right]\right\|}_{2}^{2}$$ (6)

where

$$M=\left[\begin{array}{cc} X & A+E\\ I & 0 \end{array}\right].$$

If ***A*** is in one of the classes considered in Sec. 4.1, then the matrix ***M*** has low displacement rank and we can solve the problem fast.

### 4.3 Solving Regularized Total Least Squares Problems

In many deblurring problems and other discretized problems involving integral equations of the first kind, the matrix ***A*** is so ill-conditioned that noise in the observations ***y*** is magnified in solving the STLS problem and a meaningful solution cannot be obtained.

In this case it is necessary to add a *regularization constraint* to the problem. One common regularization constraint is to restrict the size of the solution, or some linear transformation of the solution:

‖Cx‖≤u

where *u* is a given scalar and ***C*** is commonly chosen to be the identity matrix or a difference operator. If ***C*** has low displacement rank relative to ***Z***_2_ and an appropriate ${\hat{Z}}_{1}$, our algorithm can be easily modified to incorporate regularization. In this case, our problem (5) can be reformulated as

$$\underset{\alpha ,\beta ,x}{\min}{\left\|\left[\begin{array}{c} \beta \\ \alpha \\ \lambda \mathrm{Cx} \end{array}\right]\right\|}_{2}^{2}$$ (7)

where ***β*** = (***A*** + ***E***)***x*** – ***y*** and *λ*, the regularization parameter, is the Lagrange multiplier for the new constraint. Using a derivation similar to that above, the linearization of (7) results in the following problem to be solved at each step of the iteration:

$$\underset{\Delta \alpha ,\Delta x}{\min}{\left\|\left[\begin{array}{cc} X & A+E\\ I & 0\\ 0 & \lambda C \end{array}\right]\left[\begin{array}{c} \Delta \alpha \\ \Delta x \end{array}\right]+\left[\begin{array}{c} \beta \\ \alpha \\ \lambda \mathrm{Cx} \end{array}\right]\right\|}_{p}.$$

Thus, our new ***M*** matrix is the matrix ***M*** from the previous section augmented by the extra rows [**0**, *λ****C***], and the only change necessary in the algorithm is to find the generators of this matrix rather than the old one.

The displacement structure of this matrix is greatly simplified if ***C*** is upper triangular and ***Z***_1_ and ***Z***_2_ are shift-down matrices. As noted before, ***W*** is zero except for a one in the last position of the first row, and thus ***WM*** is zero except for a *λ* in the last position of the first row. Therefore, $\mathrm{WM}Z_{2}^{H}=0$, so, applying Theorem 1, we have the following result.

**Theorem 3.** If ***C*** is upper triangular and ***Z***_1_ and ***Z***_2_ are shift-down matrices, then

$$M^{H}M-Z_{2}M^{H}MZ_{2}^{H}={\left(M-N/2\right)}^{H}N+N^{H}\left(M-N/2\right)$$ (8)

and has rank 2*ρ*_1_, where *ρ*_1_ is the rank of ***N***. More generally, (8) holds whenever $\mathrm{WM}Z_{2}^{H}=0$.

## 5. Conclusions

We have derived the generators for ***M****^H^****M*** when ***M*** is any matrix of small displacement rank relative to ***Z***_1_ and ***Z***_2_. We have shown that it is inexpensive to form a Cholesky factorization of ***M****^H^****M*** whenever ***Z***_1_ is close to unitary and ***Z***_2_ is triangular and nilpotent, and we have generalized this algorithm when a regularization constraint is to be applied.
